# Comorbidities and concentration of trace elements in livers of European bison from Bieszczady Mountains (Poland)

**DOI:** 10.1038/s41598-023-31245-z

**Published:** 2023-03-15

**Authors:** Daniel Klich, Rafał Łopucki, Stanisław Kaczor, Iwona Zwolak, Anna Didkowska, Dariusz Wiącek, Wojciech Bielecki, Kajetan Perzanowski, Marlena Wojciechowska, Wanda Olech

**Affiliations:** 1 Department of Animal Genetics and Conservation, Warsaw University of Life Sciences (SGGW), Ciszewskiego 8, 02-786 Warsaw, Poland; 2 Department of Biomedicine and Environmental Research, The John Paul II Catholic University of Lublin, Konstantynów 1J, 20-708 Lublin, Poland; 3 County Veterinary Inspectorate, Młynarska 45, 38-500 Sanok, Poland; 4 Department of Food Hygiene and Public Health Protection, Institute of Veterinary Medicine, Warsaw University of Life Sciences (SGGW), Nowoursynowska 166, 02-787 Warsaw, Poland; 5 Institute of Agrophysics, Polish Academy of Sciences, Doświadczalna 4, 20-290 Lublin, Poland; 6 Department of Pathology and Veterinary Diagnostics, Institute of Veterinary Medicine, Warsaw University of Life Sciences (SGGW), Nowoursynowska 166, 02-787 Warsaw, Poland; 7 The Institute of Biological Sciences, The John Paul II Catholic University of Lublin, Konstantynów 1 H, 20-708 Lublin, Poland

**Keywords:** Environmental chemistry, Environmental impact, Diseases

## Abstract

European bison is a species for which health monitoring is essential in conservation activities. So far, little research has been carried out on the concentration of elements in this species. Most previous studies did not associate the concentration of elements with susceptibility to diseases. In this study we investigate the relationship between comorbidities in European bison and concentrations of a wide spectrum of elements in the liver. Samples were collected during the monitoring of the European bison population in Bieszczady (southeast Poland) over the 2020–2022 period. Each individual was also visually inspected by a veterinarian in the field for the presence of lesions as a part of a post-mortem examination. The animals were divided into 3 groups: group A—one type of clinical sign; group B—two types of clinical signs; group C—three or more types of clinical signs. The ICP-OES method was applied to assess the concentration of 40 elements in livers. Discriminant analysis showed clear differences between the mineral status of individuals in the groups with one, two, and at least three types of clinical signs. Detailed analysis of selected elements showed that, in the case of eight elements, there was a relationship with age, sex, or comorbidities. Cu, Se, and Zn showed significant differences in relation to comorbidities, but only Cu concentration was lower when the frequency of lesions was higher. We concluded that in research on the mineral status of the population, apart from the availability of trace elements in the environment, the health condition of the studied individuals should also be considered. However, inferring the mineral status of the population on the basis of randomly obtained samples from dead individuals may give an incomplete view of the population, especially in the case of species susceptible to diseases, such as European bison.

## Introduction

The conservation of large mammal populations in the contemporary human-transformed landscapes of Europe is a difficult and complicated task. A deficit of optimal habitats, low population sizes, low genetic variability, susceptibility to diseases, migration restrictions, and xenobiotics are just some of the more important problems faced by managers of large mammal populations^1,2^. Health condition is the key parameter used by conservationists as it coalesces the impact of these various threats. In the case of large mammals, this parameter (rather than a simple measure of the increase in animal numbers) is considered to be a useful indicator for assessing the effectiveness of conservation measures^3,4^. Unfortunately, studies of the mineral status of free-living protected species of large mammals, including the European bison, are difficult to conduct. The main problem is the difficulty in obtaining research samples from a representative group of animals, especially when the material cannot be collected ante-mortem, e.g., tissue samples from internal organs. For this reason, knowledge on the physiology of this species is based either on studies of captive individuals or on a few field samples, often collected from random individuals. Small and unscheduled collected research material makes it impossible to perform in-depth statistical analyses and draw reliable inferences about the impact of habitat factors on the health condition of protected animals.

European bison (*Bison bonasus* L.) is a species for which health monitoring is essential in conservation activities^5–8^. Due to the low genetic variability of the European bison, even large free-living herds struggle with health problems, and sometimes it is necessary to implement restrictive measures to eliminate sick individuals (e.g. Ref.^9^). However, the health condition of the European bison is influenced not only by genetic factors but also by the quality of habitats, the possibility of obtaining an appropriate amount of vital micro and macro elements from the environment, as well as the ability to avoid poisoning with undesirable elements^10,11^. It is known that the mineral status of the body is strongly related to the health condition, and deficiencies of elements or heavy metal poisoning may, directly and indirectly, affect resistance to various diseases, including parasitic infestations^12^. The molecular mechanisms responsible for the biological function of minerals in immunity are primarily related to the distinct role of these elements in the activity of multiple enzyme systems and the regulation of gene expression^13^.

Mineral concentrations in European bison have rarely been studied, and little research has been conducted to link the European bison’s susceptibility to diseases with concentrations of elements. One example of such research is the paper by Dymnicka et al.^14^, who found a lower level of Fe in the blood serum of animals with balanoposthitis. Moreover, Hoby et al.^15^ found elevated iodine and lowered manganese levels in the blood of European bison with digital dermatitis lesions. However, Dziaba et al.^16^ found no difference between healthy European bison and those with parasitic invasion (*Fasciola hepatica*) and lesions indicating necrotic balanoposthitis in the concentration of selected elements in the serum. In other papers, the concentrations of selected elements are usually described but without a detailed connection to the health status of the studied species. For example, Kośla et al.^17^ found that calcium concentration in selected tissues of European bison from Białowieska Forest was higher than in other bovids (wild and domestic). Se deficiency was found in European bison in Białowieska Forest, which presented the lowest level in livers compared to other populations in eastern Poland^10,11,18,19^. Some individuals in Białowieska Forest also presented a deficiency of Co, Cu, Mn, Na and Zn^10,14^. Moreover, even lower Cu and Na concentrations were found in Knyszyńska Forest compared to Białowieska Forest, which was probably an effect of the animals feeding on crops^11^. On the other hand, European bison in the Bieszczady Mountains presented the highest Ba, Ca, Cd and Se concentrations compared to other populations in eastern Poland^11^. Moreover, extremely high Cd concentrations were found in some animals in the Bieszczady Mountains.

Taking the above into account, we attempted to assess the relationship between the concentrations of micro and macro elements and susceptibility to diseases in European bison. The study was carried out on European bison in the Bieszczady Mountains, and researchers found that these animals seem to be highly susceptible to certain health disorders, including infectious diseases, such as bovine tuberculosis^6^ or thelasiosis, which causes blindness in various bovine species^20^. In recent years, these two diseases have forced the elimination of dozens of European bison in this area. In the Bieszczady Mountains, an increased prevalence of *Neospora caninum* antibodies^21^ and Schmallenberg virus^22^ was also found more often than in other Polish populations. In this area, the first case of *Ashwortius sidemi* (Nematoda, Trichostrongylidae, a new parasite of the European bison) was found in Poland^23^.

The aim of this study was to attempt a comprehensive investigation of the relationship between comorbidities in European bison and concentrations of a wide spectrum of elements in the liver. We used a uniquely large research sample: 63 samples of livers collected from individuals for which autopsy protocols were prepared at the same time. In this paper, we hypothesize that, depending on the degree of disease burden, the hepatic mineral status of the studied individuals varies, and significant differences in the concentrations of specific vital elements are observed.

## Methods

All experimental protocols (including culling of European bison) were approved by the General Directorate for Environmental Protection in Poland, based on the Act of 16 April 2004 on The Protection of Nature. All methods were carried out in accordance with relevant guidelines and regulations. Namely, the collection and storage of samples of dead individuals for the study were based on the decision of the Regional Director of Environmental Protection in Warsaw. All methods are reported in accordance with ARRIVE guidelines.

### Sample and data collection

Data and samples were collected over the period May 2020 to March 2022 (all months, except January and July) during the monitoring of the European bison population in Bieszczady (southeast Poland). 61 of the 63 individuals were eliminated as part of the fight against thelasiosis. Two animals were found dead. This culling of European bison was legal because a relevant permit was obtained in advance from the General Directorate for Environmental Protection in Poland. All other legal issues related to these activities are described in Klich et al.^2^. The criterion for selection of European bison for elimination were clear changes in the eyeball, opaqueness of the eyeball, or blindness. Animals were eliminated by a shot from a hunting weapon in the area of the spine, without damaging the internal organs. Pharmacological agents were not used. Data about the age, sex, and location of each animal (using a GPS receiver) were collected. As a part of the *post-mortem* examination, each individual was also visually inspected for the presence of lesions by veterinarians in the field. The animals underwent autopsy according to the established protocol. The autopsies were carried out by a team of three veterinarians. Numerous anatomopathological lesions indicating diseases were found in these European bison; usually, there was more than one type of lesion per individual. The lesions found were: (1) lung nematodes; (2) lesions indicating thelasiosis (congestion of the conjunctival sac, keratitis and corneal opacity, anophthalmia, formation of erosion and ulcers on the eye, blindness); (3) lesions indicating previous pneumonia; (4) lesions indicating previous enteritis; (5) kidney cysts; (6) tuberculosis-like lesions (TBL); (7) multiple abscesses on the whole carcass; (8) lesions indicating necrotic dermatitis; (9) lesions indicating necrotic balanoposthitis; (10) tracheal bronchi lesions.

**Figure 1:**
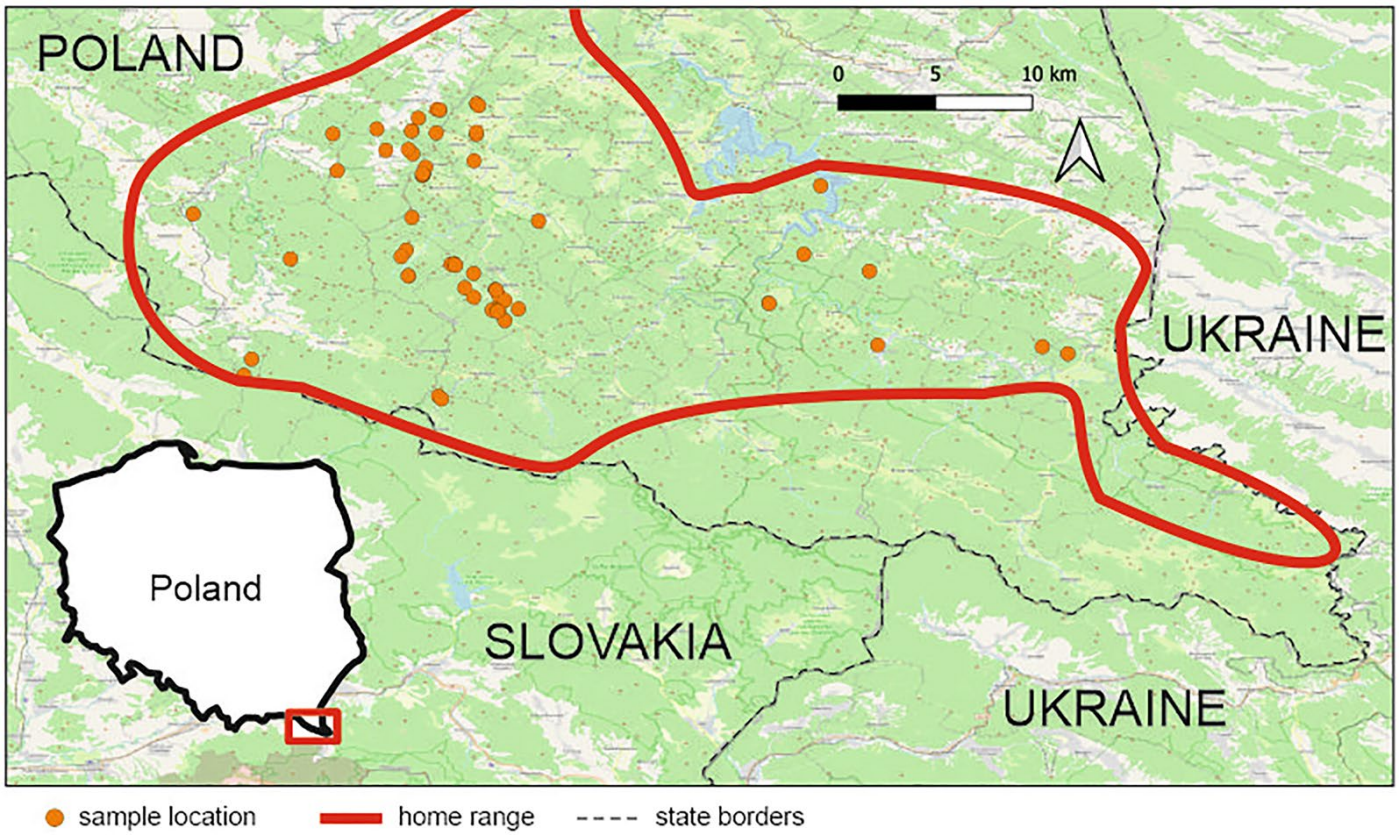
Study area—location of the eliminated European bison that were sampled for this study. The figure was generated in Quantum GIS v.3.4.5 (https://qgis.org) and CorelDRAW Standard 2020 (https://www.corel.com).

During the veterinary inspection, liver samples were collected, cooled in a portable fridge, transported to the laboratory and stored at – 20 °C until analysis. Liver samples were taken from the central part of the right lobe. In total, liver samples were collected from 27 females (aged from 0.5 to 18 years) and 36 males (aged from 3 to 20 years). Pregnancy was not observed in any of the cows. We collected a total of 63 samples from various locations within the Bieszczady Mountains, covering both the western and eastern edges of the European bison’s home range there (Fig. 1). Lesions were found in 62 of the 63 animals.

### ICP-OES: sample preparation and analysis

The laboratory procedure for analysis of liver samples by ICP-OES spectrometry has already been described by Klich et al.^11^. Here we followed the same procedure. Briefly (based on Klich et al.’s^11^ description), 500 mg lyophilized liver sample was weighed with an accuracy of 1 mg. Then, 10 mL of concentrated analytical grade HNO_3_ (Sigma Aldrich, Germany) was added and mineralization was carried out using a microwave digestion system (Speedwave, Berghof, Germany). Next, this clear solution was transferred to a 50 mL volumetric flask, which was then filled with demineralized water and analyzed using ICP-OES spectrometry (Thermo Fisher Scientific iCAP6500). The following instrumental settings were used: RF generator power of 1150 W, RF generator frequency of 27.12 MHz, coolant gas flow rate of 16 L min^−1^, carrier gas flow rate of 0.65 L min^−1^, auxiliary gas flow rate of 0.4 L min^−1^, max integration time of 15 s, pump rate of 50 rpm, axial viewing configuration, 3 replicates, and a 20 s flush time.

Similar to Klich et al.’s^11^ analytical procedure, the following multielement stock solutions (Inorganic Ventures) were used as standards:

1. CCS-4: Al, As, Ba, Be, Bi, Ca, Cs, Ga, In, K, Li, Mg, Na, Rb, Se, Sr 100 mg/L in 7% HNO_3_.
2. CCS-5: B, Ge, Hf, Mo, Nb, P, Re, S, Sb, Si, Sn, Ta, Ti, W, Zr 100 mg/L in 7% HNO_3_ and 1% HF.
3. CCS-6: Ag, Cd, Co, Cr, Cu, Fe, Hg, Mn, Ni, Pb, Tl, V, Zn 100 mg/L in 7% HNO_3_.

Similarly to Klich et al.^11^, to calculate the percentage recovery, three randomly selected samples that were individually spiked with known amounts of the analytical standards were used as positive controls (Supplementary Table S1). Additionally, a certified reference material, NIST Bovine liver 1577c (Sigma Aldrich, Germany), was analyzed in order to check the correctness of the method^11^. The accuracy of the method was estimated on the basis of a multielement certified reference material, Periodic table mix 1 TraceCERT (Sigma Aldrich, Germany). A multielement standard sample was measured every dozen or so samples (Supplementary Table S2).

#### Data elaboration and statistics

Animals were grouped in terms of the number of identified pathological changes because some types of lesions were usually found too rarely, or they occurred in almost all individuals. It should be noted that the liver samples came from individuals eliminated from the population mainly due to visible clinical signs; therefore, theoretically, each of them should have had lesions in the post-mortem examination, so it was not possible to make a comparison with healthy individuals. Considering that all the studied European bison showed various lesions, the animals were divided into 3 groups, irrespective of the type of lesions: group A—one type of clinical sign; group B—two types of clinical signs; group C—three or more types of clinical signs. Each individual was thus assigned to a comorbidities category. Using these categories (groups), we performed general and detailed analyses of the differences in the mineral status of the studied individuals.

The general analysis included the results of the concentrations of all 40 determined elements and was carried out to express the general differences in the mineral status of the studied groups of individuals (A, B and C). To do this, discriminant analysis was used, where groups A, B and C were used as grouping variables, and a concentration of all studied elements were used as explanatory variables. Prior to this analysis, the data were normalized by Box-Cox transformation. Statistical analysis was performed using Statistica software v13.3 (TIBCO Software Inc.).

The detailed analysis was conducted for 19 selected elements, i.e., the basic micro and macro elements that are most often described in the literature: Al, As, Ca, Cd, Co, Cu, Fe, Li, Hg, Mg, Mn, Mo, Ni, Pb, Se, Sn, Ti, V, and Zn. Three types of statistical tests were performed with IBM SPSS v24.0 (Armonk, New York):

1. The element concentration patterns were identified with Pearson’s correlation, where all 19 elements were correlated with each other using all 63 samples.
2. Linear models were used to analyze the linear dependence of each element on disease susceptibility (as above) and the sex and age of the animals. Prior to this analysis, the dependent variables (concentration of a given element) that were not normally distributed were normalized by Box-Cox transformation. A general linear model (for variables that showed a normal distribution) or a generalized linear model (for variables for which the transformation failed) was used. 19 linear models for each element were built separately using 62 samples (only animals with lesions).
3. The effect of selected pathological changes in European bison (using 62 samples) on three selected elements (i.e. Cu, Se and Zn, which were statistically explained by comorbidities) was verified using linear models (general linear models for Se, and generalized linear model for Cu and Zn). The dependent variable in each model was the concentration of a given element. The explanatory variables were (a) the presence of lung nematodes (LUNG_NEM); (b) the presence of lesions indicating previous pneumonia (PNEUMONIA); (c) the presence of all other lesions together, except for lesions indicating thelasiosis (OTHER). The lesions were combined in this analysis because each of them was rarely present in the studied animals. We did not include lesions indicating thelasiosis because almost all the animals suffered from this disease.

## Results

Individual lesions occurred with different frequency in the studied European bison. Most often found were lesions indicating thelasiosis (97%, in 61 out of 63 ind.), the presence of lung nematodes (41%, in 26 out of 63 ind.) and lesions indicating previous pneumonia (22%, in 14 out of 63 ind.). Other types of anatomopathological lesions were found in only one to three individuals (Supplementary Table S3).

**Figure 2:**
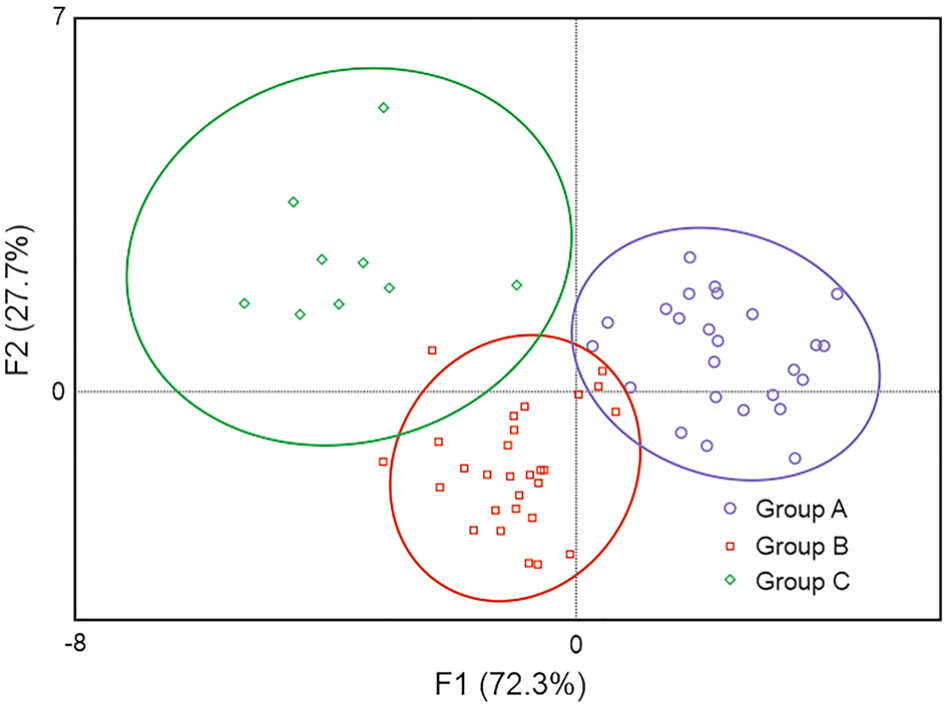
Plot of functions F1 and F2, which were used in the discriminant analysis of the mineral status of the three European bison groups with different number of lesions (group A—one type of clinical sign; group B—two types of clinical signs; group C—three or more types of clinical signs) based on ICP-OES analysis of 40 elements in liver samples. The cumulative discrimination value of functions F1 and F2 is 100%. The figure was generated in Statistica v13.3 (https://www.statsoft.pl/statistica_13/) and CorelDRAW Standard 2020 (https://www.corel.com).

Discriminant analysis showed clear differences in the mineral status in the groups of individuals with one, two and three types of clinical signs, even without considering the type of lesion (Fig. 2). Comorbidities clearly separated the European bison in terms of the concentration of elements. European bison of group C (with three or more types of clinical signs) were distinct from the other groups (A and B), but also group B (animals with two types of clinical signs) differed from group A (European bison with one type of clinical sign). Therefore, each subsequent disease causes a clear multidimensional effect in the hepatic concentrations of the studied elements. The analyzed data can be described by means of two discriminant functions (F1 and F2), which describe 100% of the variability (Fig. 2, Table 1). The concentration of elements is presented in Supplementary Table S4.

**Table 1:** Chi-square tests for the particular discriminant functions (F1 and F2) obtained for the 40 trace elements that were analyzed in the liver samples of European bison in the three groups (A, B, C).

Discriminant functions	Eigenvalue	R	Wilk’s lambda	Chi-square	df	p
F1	4.89	0.91	0.059	111.77	80	0.011
F2	1.87	0.81	0.348	41.69	39	0.354

In total, 53 statistically significant correlations between the analyzed elements were found (Supplementary table S5). Positive relations predominated (37 positive relations and 16 negative relations). Out of the 19 analyzed elements, only Fe did not show a linear relationship with any of the other elements. Most often, Ca or Se correlated with other elements, but both correlated with six elements (Ca correlated with Cd, Mg, Ni, Se, V and Zn; Se correlated with As, Ca, Cd, Hg, Li, and Mg). The correlations were weak, with the value of Pearson’s r not exceeding 0.5 in any of them. The strongest relationships occurred in pairs: Al–Zn (r = 0.480), As–Se (r = − 0.457) and Cd–Se (r = 0.459).

**Figure 3:**
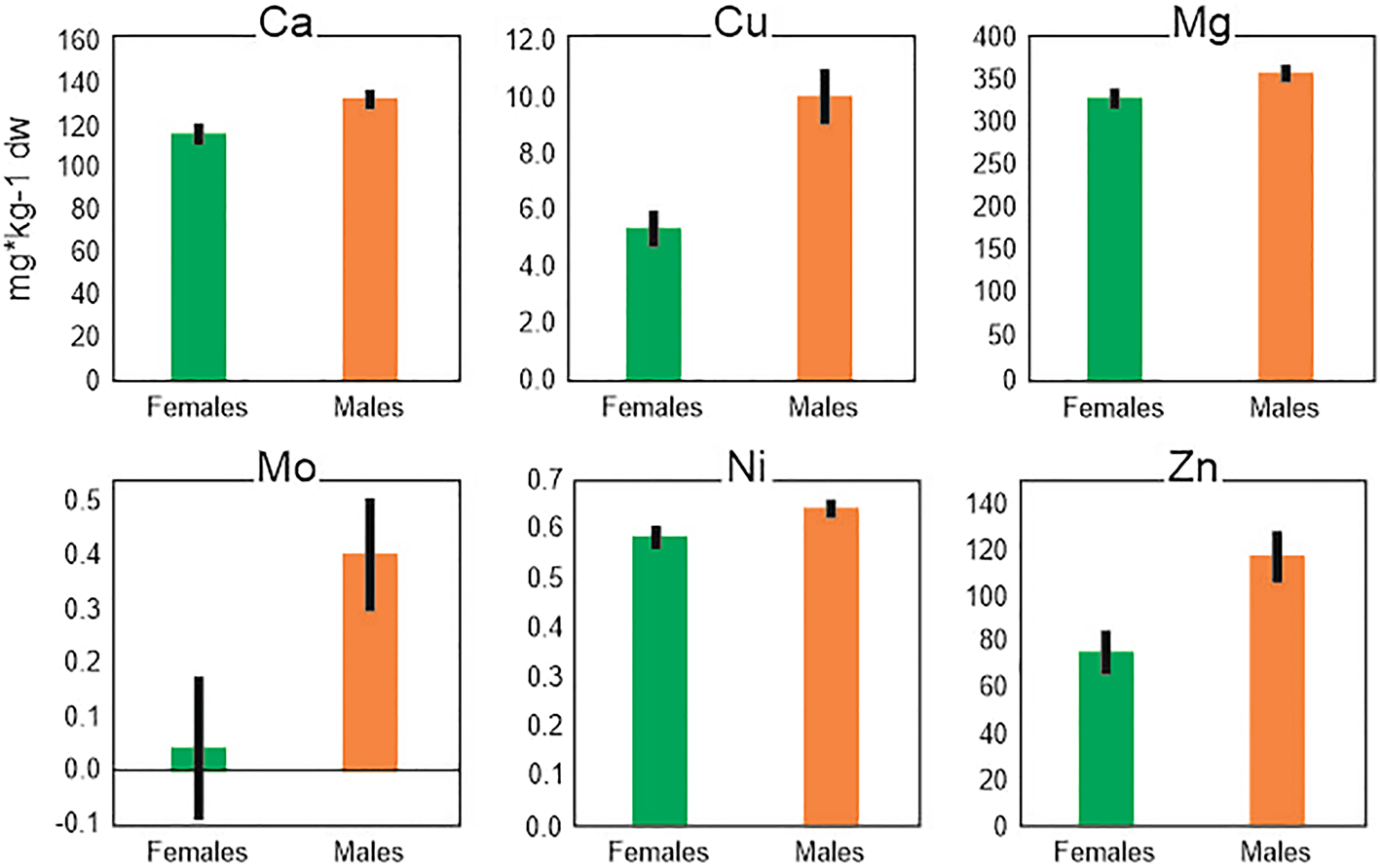
Mean (± SE) hepatic concentration of Ca, Cu, Mg, Mo, Ni, Zn with regard to sex. All pairwise comparisons were statistically significant (p < 0.05) in the general (for Ca, Mg, Mn, Mo and Ni) or generalized (for Cu and Zn) linear models (measurement units do not apply to Mo, which was BOX Cox transformed). The figure was generated in SPSS v24.0 (https://www.ibm.com/products/spss-statistics) and CorelDRAW Standard 2020 (https://www.corel.com).

**Figure 4:**
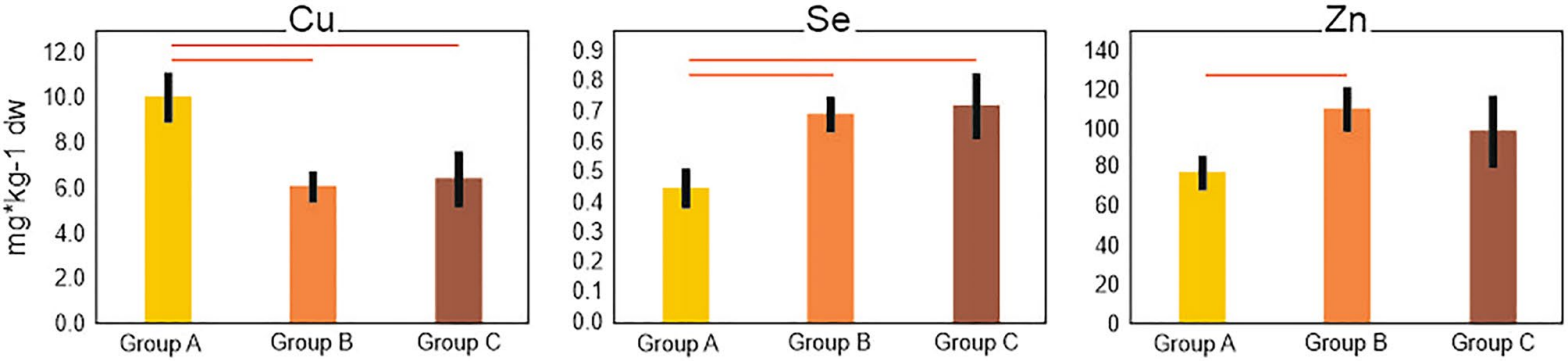
Mean (± SE) hepatic concentration of Cu, Se, and Zn in the three groups of European bison regarding presence of comorbidities (for details, see methods). Red horizontal lines indicate statistical significance of the pairwise comparisons (p < 0.05) in the general (Se) or generalized (for Cu and Zn) linear models. The figure was generated in SPSS v24.0 (https://www.ibm.com/products/spss-statistics) and CorelDRAW Standard 2020 (https://www.corel.com).

Detailed analysis of selected elements showed that, in the case of eight elements, a relationship with age, sex or comorbidities was found. Only two elements (Cd and Ni) correlated with the age of the animals (Supplementary Table S6). However, only the cadmium concentration showed a positive relation with age. Six elements differed with sex (Ca, Cu, Mg, Mo, Ni, Zn), and females always had lower concentrations of these elements than males (Fig. 3). Out of the 19 analyzed elements, three showed significant differences in relation to comorbidities: Cu, Se, and Zn (Fig. 4). European bison with a lower frequency of various lesions had a higher concentration of Cu. In another two cases (Se, Zn), the more numerous the lesions (groups B and C), the higher the concentrations of these elements. Nevertheless, in the case of Zn, group A differed significantly from group B but not from group C; in the case of Cu and Se, group A differed statistically from both groups B and C (Fig. 4).

Linear model analysis of particular types of lesions in relation to the concentration of elements in the liver of European bison showed no statistically significant effect for Cu (Table 2). In the case of Zn, the OTHER category, which includes different types of changes, showed a significant effect in the model. In the case of Se, only changes indicating lung nematodes significantly differentiated European bison with regard to the concentration of this element in the liver (Table 2).

**Table 2:** Effects of selected lesions on Cu, Se and Zn concentrations in the livers of European bison in the general linear model (for Se) and the generalized linear model (for Cu and Zn).

Source	Cu		Se		Zn	
	Wald chi^2^	p	F	p	Wald chi^2^	p
Intercept	267.246	< 0.001	136.725	< 0.001	2306.006	< 0.001
LUNG_NEM	0.836	0.360	7.549	0.008	0.09	0.736
PNEUMONIA	2.334	0.127	0.078	0.782	0.00	0.996
OTHER	2.596	0.107	3.478	0.067	20.750	< 0.001

## Discussion

In this paper, using relatively large and unique research material, we have shown significant deficits in the concentration of key elements, as well as interrelationships between the mineral status and comorbidities in the studied animals. Below, we try to show which ecological and physiological mechanisms may be responsible for these results.

The liver is a major storage organ of the labile pool of essential trace elements in the body^24^, and it is where the synthesis of most acute-phase proteins, i.e., metal transport proteins^25^, occurs. The concentration of certain elements in the liver reflects their levels and availability in the environment. As a result of inflammation and infectious diseases, however, there is a redistribution of elements between tissues and blood that is triggered by infection-induced synthesis of metal-binding (transport) proteins, including acute-phase proteins and antagonism between trace elements^26–30^. In our study, we are probably dealing with a combined effect of these processes, regarding both the content (availability) of certain elements in the environment as well as physiological disorders caused by disease states and element antagonisms.

In comparison to the reference values for cattle^31^, the observed average liver levels of Cu and Se indicates their deficiency in the body. One of the main reasons for the deficiency of these microelements in the tissues of ruminants is poor bioavailability and/or a low level of these microelements in the soil where these animals live^32^. In Poland, the average level of Se in the soil is 0.27 mg kg^−1^ d.w.^33^, while according to the paper of Gupta & Gupta^34^, Se level within the range of 0.1–0.6 mg kg^−1^ is considered a deficit. Hence, Se deficiency is often observed in the tissues of free-living ruminants in Poland, including European bison^10,11,35,36^. Furthermore, high levels of S and Mo in the soil and diet (despite the appropriate level of Cu) can significantly reduce Cu absorption in the intestines of ruminants, thus inducing a secondary deficiency of this microelement^37,38^. Applications of N and P can also lead to the dilution of Cu in crop plants’ tissues^39^. Moreover, Cd can directly compete with Cu for absorption sites^40,41^. In the Bieszczady Mountains, high Cd concentration was found, and European bison consume crops in this area^42^.

One of the main signs of Se and Cu deficiency in cattle is the susceptibility of these animals to infections^43–45^. In this work, the studied group of animals had both Se and Cu deficiency and at least one type of disease symptom (61 out of 63 animals presented Cu deficiency, and 53 of the 63 animals presented Se deficiency). It could therefore be suspected that this simultaneous deficiency of Se and Cu caused deterioration of the European bison’s health. The role of Cu and Se in the immune system is, among others, antioxidant: Cu functions as a cofactor for Cu/Zn-superoxidase dismutase (Cu/Zn-SOD) and Se is essential in the active center of glutathione peroxidases (GPx)^46,47^. Furthermore, another Cu-protein, cytochrome C oxidase, is an integral part of the oxygen chain and is involved in ATP formation. Cu deficiency in cattle is known to reduce the activity of Cu/Zn-SOD and cytochrome C oxidase in leukocytes^48^. Other immune functions of Cu and Se include their role in T-cell proliferation and differentiation (Se and Cu), and their participation in direct antimicrobial effect (Cu)^46^. Additionally, essential trace elements (Cu, Zn) are also integral components of ceruloplasmin and Zn-binding metallothionein (acute-phase proteins of the Cu-protein type), the latter of which is produced during the acute phase of infectious disease^26^.

In the examined tissue, as comorbidity increases (two or more diseases per individual), concentrations of Zn and Se increase, but the level of Cu decreases. Similar results, i.e., an increase in Zn level during disease, was observed in adjuvant arthritis rats^30^, in mice infected with Coxsackievirus B3^27^, and in mice with sepsis^49^. Rhodes et al.^50^ found that malabsorption of Cu in American bison could be caused by increased absorption of Se, Mo or Zn, as well as parasite invasions. These authors concluded that imbalance of trace elements and parasitic infestation contributed to animals’ decreased weights and birth rates. A characteristic feature of most acute infectious diseases (regardless of the factor causing them) is a decrease in Zn level and an increase in Cu level in the serum (increase in Cu/Zn ratio)^28,51^. Here, in the studied European bison, the increase in Zn level in the liver (statistically related to comorbidities in European bison) was probably the result of the mobilization of this element from the serum to the liver, whereas the decrease in Cu in the liver was related to an increased release of Cu from this tissue into the serum. Chronic inflammation promotes the redistribution of Zn from the plasma to the liver, which is explained, among others, by the inflammatory factor-induced expression of Zn importer (ZIP14) and the synthesis of metallothionein in the liver tissue^49,52^. Moreover, excessive ceruloplasmin synthesis during inflammation can drain Cu from other tissues, especially from the liver, which is the main site of this protein’s synthesis^53^.

In studied European bison, higher concentrations of Se in the liver were also observed in individuals with a greater number of pathological changes compared to individuals with only one type of a lesion. This result suggests that the metabolism of Se can be affected by an increase in the number of lesions because the accumulation of Se in the liver was observed with a greater number of lesions. The data in the literature concerning the influence of disease states (infections) on Se concentration in the liver are quite ambiguous^54–57^. It can be speculated, however, that the increased Se accumulation observed in our study could be a result of changes that take place in the body during inflammation. Notably, during the inflammation that accompanies infections, the permeability of blood vessels increases, which could lead to increased Se transfer from circulation to tissues^47^.

The results presented in this paper, apart from the cognitive aspect, also have a practical dimension because they clearly show that when undertaking research on the mineral status of a given animal population, the key element is to take into account the health condition of the studied individuals. It is true that there are still large gaps in the knowledge, and more research is needed to reliably conclude on the health conditions of protected species. Unfortunately, the possibilities of performing such tests are often limited, therefore the obtained results should be approached with great caution. Inferring the mineral status of a given animal population on the basis of randomly obtained samples from dead individuals may give an incomplete and biased view of the population, especially in the case of species susceptible to diseases, such as European bison. In some cases, however, as an indicator of the mineral status of wild animals, the analysis of micro and macro elements contents in hair, sampled in vivo, can be useful^58^. Nevertheless, the results of our research show that the mineral status of an individual in a given population can be significantly influenced by even a single ailment/disease. This shows the importance of professional and thorough veterinary inspection of the individual from which samples are taken for laboratory analysis. At the same time, it shows the need for more interdisciplinarity research on species such as European bison that combines ecological, conservation, physiological and veterinary issues.

## Supplementary Information

Supplementary Table S1.

Supplementary Table S2.

Supplementary Table S3.

Supplementary Table S4.

Supplementary Table S5.

Supplementary Table S6.

## Data Availability

The datasets generated during the current study are available from the corresponding author on reasonable request.
