# Supplementary material for: Comorbidities and concentration of trace elements in livers of European bison from Bieszczady Mountains (Poland)
Source: Sci Rep. 2023 Mar 15;13:4332. doi: 10.1038/s41598-023-31245-z (PMC10017800; doi:10.1038/s41598-023-31245-z)
Supplement: Supplementary file 1 — Supplementary Table S1. [file 41598_2023_31245_MOESM1_ESM.docx]

Table S1. Validation of the analytical method used in this study: linearity (the ability of the method to obtain test results proportional to the concentration of the analyte), limit of detection (LOD), and recoveries for the studied elements.

| Element | Coefficient of correlation R | Limit of detection LOD [mg/kg] | Recovery [%] |
| --- | --- | --- | --- |
|  |  |  |  |
| Al | 0.9999 | 0.028 | 106 |
| As | 0.9998 | 0.017 | 96 |
| B | 0.9998 | 0.055 | 112 |
| Ba | 0.9998 | 0.018 | 108 |
| Be | 0.9997 | 0.012 | 97 |
| Bi | 0.9996 | 0.065 | 106 |
| Ca | 0.9994 | 0.008 | 107 |
| Cd | 0.9999 | 0.003 | 97 |
| Co | 0.9999 | 0.002 | 98 |
| Cr | 0.9998 | 0.002 | 105 |
| Cu | 0.9999 | 0.004 | 104 |
| Fe | 0.9998 | 0.014 | 109 |
| Hg | 0.9998 | 0.040 | 94 |
| In | 0.9993 | 0.122 | 92 |
| K | 0.9995 | 0.016 | 105 |
| Li | 0.9990 | 0.008 | 103 |
| Mg | 0.9987 | 0.010 | 108 |
| Mn | 0.9998 | 0.004 | 97 |
| Mo | 0.9999 | 0.013 | 98 |
| Na | 0.9991 | 0.031 | 108 |
| Nb | 0.9998 | 0.045 | 97 |
| Ni | 1.0000 | 0.003 | 95 |
| P | 0.9999 | 0.052 | 107 |
| Pb | 0.9999 | 0.008 | 96 |
| Rb | 0.9999 | 0.018 | 98 |
| S | 0.9997 | 0.014 | 105 |
| Sb | 0.9998 | 0.015 | 96 |
| Se | 0.9999 | 0.012 | 96 |
| Si | 0.9993 | 0.010 | 94 |
| Sn | 09997 | 0.015 | 98 |
| Sr | 0.9998 | 0.008 | 104 |
| Ta | 0.9995 | 0.074 | 94 |
| Ti | 0.9999 | 0.009 | 98 |
| Tl | 0.9999 | 0.068 | 103 |
| V | 1.0000 | 0.008 | 98 |
| W | 0.9999 | 0.009 | 98 |
| Zn | 0.9999 | 0.011 | 104 |
| Zr | 0.9999 | 0.010 | 103 |

Due to the fact that the element Al was not certified in the reference material, the recoveries for this element were calculated using the standard addition method. Single Al standard element was used for measurements (MsAl - 10ppm, 10 ug/mL, Inorganic Ventures, USA). Different amounts of Al standard (0.5-10 ug/mL) were added to six liver samples and then mineralized. The recoveries ranged from 95.2% to 118.4% with a mean of 110.8%.
