# Supplementary material for: Comorbidities and concentration of trace elements in livers of European bison from Bieszczady Mountains (Poland)
Source: Sci Rep. 2023 Mar 15;13:4332. doi: 10.1038/s41598-023-31245-z (PMC10017800; doi:10.1038/s41598-023-31245-z)
Supplement: Supplementary file 2 — Supplementary Table S2. [file 41598_2023_31245_MOESM2_ESM.docx]

Table S2. In order to check the correctness of the method, the certified reference material was analyzed– NIST Bovine liver 1577c (Sigma Aldrich, Germany). The results of material analysis are presented in the Table . The accuracy of the method was estimate on the basis multielement certified reference material Periodic table mix 1 TraceCERT (Sigma Aldrich, Germany). The accuracy of the method was checked during the measurements. A multielement standard sample was measured every dozen or so sample.

| **Element** | Certified value – NIST Bovine liver 1577c  [mg/kg] | Measured value [mg/kg] | Recovery (%) |
| --- | --- | --- | --- |
| Al | - | 22.35 | - |
| As | 19.6 | 18.90 | 96 |
| Ba | - | 0.2870 | - |
| Be | - | 0.0113 | - |
| Ca | 131 | 142.1 | 108 |
| Cd | 97.0 | 95.42 | 98 |
| Co | 0.30 | 0.2892 | 96 |
| Cr | 53.0 | 58.04 | 109 |
| Cu | 275.2 | 281.3 | 102 |
| Fe | 197.94 | 216.0 | 109 |
| Hg | - | 0.2241 | - |
| K | - | 7826 | - |
| Li | - | 0.0185 | - |
| Mg | 620 | 653.6 | 105 |
| Mn | 10.46 | 9.873 | 94 |
| Mo | 3.30 | 3.280 | 99 |
| Na | 2033 | 2264 | 111 |
| Ni | 44.5 | 43.14 | 97 |
| Pb | 62.8 | 61.22 | 97 |
| S | 7490 | 7740 | 103 |
| Sb | - | 0.1643 | - |
| Se | 2.031 | 2.015 | 99 |
| Si | - | 1.481 | - |
| Sn | - | 0.1780 | - |
| Ti | - | 0.2423 | - |
| V | 8.17 | 8.021 | 98 |
| Zn | 181.1 | 184.5 | 102 |
