# Supplementary material for: Comorbidities and concentration of trace elements in livers of European bison from Bieszczady Mountains (Poland)
Source: Sci Rep. 2023 Mar 15;13:4332. doi: 10.1038/s41598-023-31245-z (PMC10017800; doi:10.1038/s41598-023-31245-z)
Supplement: Supplementary file 3 — Supplementary Table S3. [file 41598_2023_31245_MOESM3_ESM.docx]

Table S3. Age and sex of animals and lesions found in each animal: 1) lung nematodes (NEM), 2) lesions indicating telasiosis (TEL), 3) lesions indicating a previous pneumonia (PNE), 4) lesions indicating a previous enteritis (ENT), 5) kidney cysts (CYS), 6) tuberculosis-like lesions (TBL) 7) multiple abscesses on the whole carcass (ABS), 8) lesions indicating a necrotic dermatitis (DER), 9) lesions indicating necrotic posthitis (POS), 10) tracheal bronchi lesions (BRO)

| Sex | Age (years) | NEM | TEL | PNE | ENT | CYS | TBL | ABS | DER | POS | BRO |
| --- | --- | --- | --- | --- | --- | --- | --- | --- | --- | --- | --- |
| F | 0.5 |  | 1 |  |  |  |  |  |  |  |  |
| F | 5 |  |  | 1 |  |  |  |  |  |  |  |
| F | 6 |  | 1 |  |  |  |  |  |  |  |  |
| F | 6 | 1 | 1 | 1 |  |  |  | 1 |  |  |  |
| F | 6 |  | 1 |  |  |  |  |  |  |  |  |
| F | 7 | 1 | 1 |  |  |  |  |  |  |  |  |
| F | 8 | 1 | 1 | 1 |  |  |  |  |  |  |  |
| F | 8 | 1 | 1 |  |  |  |  |  |  |  |  |
| F | 8 | 1 | 1 |  |  |  |  |  |  |  |  |
| F | 9 |  | 1 |  |  |  |  |  |  |  |  |
| F | 9 |  | 1 |  |  | 1 |  |  |  |  |  |
| F | 10 | 1 | 1 |  |  |  |  |  |  |  |  |
| F | 10 |  | 1 |  |  |  |  |  |  |  |  |
| F | 10 | 1 | 1 |  |  |  |  |  |  |  |  |
| F | 11 |  | 1 |  |  |  |  |  |  |  |  |
| F | 12 |  | 1 |  |  |  |  |  |  |  |  |
| F | 12 |  | 1 | 1 |  |  |  |  |  |  |  |
| F | 12 | 1 | 1 |  |  |  |  |  |  |  |  |
| F | 12 |  | 1 |  |  |  |  |  |  |  |  |
| F | 12 |  | 1 |  |  |  |  |  |  |  |  |
| F | 15 |  | 1 |  |  |  |  |  |  |  |  |
| F | 15 |  | 1 | 1 |  |  |  |  |  |  |  |
| F | 16 | 1 | 1 |  |  |  |  |  |  |  |  |
| F | 16 |  | 1 |  |  | 1 |  |  |  |  |  |
| F | 16 |  | 1 |  |  | 1 |  |  |  |  |  |
| F | 16 |  | 1 |  |  |  |  |  |  |  |  |
| F | 18 |  | 1 |  |  |  |  |  |  |  |  |
| M | 3 | 1 | 1 | 1 |  |  |  |  |  |  |  |
| M | 6 |  | 1 |  | 1 |  |  |  |  |  |  |
| M | 6 | 1 | 1 |  |  |  |  |  |  |  |  |
| M | 6 |  | 1 |  |  |  |  |  |  |  |  |
| M | 6 |  | 1 |  |  |  |  |  |  |  |  |
| M | 8 | 1 | 1 |  |  |  |  |  |  |  |  |
| M | 9 | 1 | 1 |  |  |  |  |  | 1 |  |  |
| M | 10 |  | 1 |  |  |  |  |  |  |  |  |
| M | 10 |  | 1 | 1 |  |  |  |  |  |  |  |
| M | 12 | 1 | 1 |  |  |  |  |  |  |  |  |
| M | 12 | 1 | 1 | 1 |  |  |  |  |  |  |  |
| M | 12 | 1 | 1 | 1 |  |  |  |  |  |  |  |
| M | 12 |  | 1 |  |  |  |  |  | 1 |  |  |
| M | 12 |  | 1 |  |  |  |  |  |  |  |  |
| M | 13 | 1 | 1 | 1 |  |  |  |  |  |  |  |
| M | 13 | 1 | 1 |  |  |  |  |  |  |  |  |
| M | 13 | 1 | 1 |  |  |  |  |  |  |  |  |
| M | 13 |  | 1 |  |  |  |  |  |  |  |  |
| M | 14 | 1 | 1 |  |  |  |  |  |  |  |  |
| M | 14 | 1 | 1 | 1 |  |  | 1 |  |  |  |  |
| M | 14 |  | 1 | 1 |  |  |  |  |  |  |  |
| M | 14 |  | 1 |  |  |  |  |  |  |  |  |
| M | 15 |  | 1 |  |  |  |  |  |  |  |  |
| M | 15 |  | 1 |  |  |  |  |  |  |  |  |
| M | 15 | 1 | 1 | 1 |  |  |  |  |  |  | 1 |
| M | 16 | 1 | 1 |  |  |  |  |  |  |  |  |
| M | 16 |  | 1 | 1 |  |  |  |  |  |  |  |
| M | 17 |  | 1 |  |  |  |  |  |  | 1 |  |
| M | 17 | 1 | 1 |  |  |  |  |  |  |  |  |
| M | 18 |  | 1 |  |  |  |  |  |  |  |  |
| M | 18 |  | 1 |  |  |  |  |  |  |  |  |
| M | 18 |  | 1 |  |  |  |  |  |  |  |  |
| M | 18 |  | 1 |  |  |  |  |  |  |  |  |
| M | 19 | 1 | 1 |  |  |  |  |  |  |  |  |
| M | 20 | 1 | 1 |  |  |  |  |  |  |  |  |
