# Supplementary material for: Comorbidities and concentration of trace elements in livers of European bison from Bieszczady Mountains (Poland)
Source: Sci Rep. 2023 Mar 15;13:4332. doi: 10.1038/s41598-023-31245-z (PMC10017800; doi:10.1038/s41598-023-31245-z)
Supplement: Supplementary file 4 — Supplementary Table S4. [file 41598_2023_31245_MOESM4_ESM.docx]

Table S4. Concentration of elements in livers of European bison (mg*kg^-1^ dw)

|  | Mean | Minimum | Maximum | Median |
| --- | --- | --- | --- | --- |
| Al | 6.918672 | 2.590667 | 17.96333 | 6.543667 |
| As | 0.282572 | 0.105133 | 0.520767 | 0.275733 |
| B | 3.702579 | 2.403667 | 4.475667 | 3.742667 |
| Ba | 0.127685 | 0.0154 | 1.598333 | 0.099633 |
| Be | 0.022835 | 0.018 | 0.034667 | 0.022667 |
| Bi | 0.546177 | 0.195133 | 0.9599 | 0.542933 |
| Ca | 125.6631 | 83.31333 | 195.3 | 128.3 |
| Cd | 2.496386 | 0.045433 | 21.96333 | 2.080667 |
| Co | 0.109986 | 0.036433 | 0.1689 | 0.110133 |
| Cr | 1.289385 | 0.7844 | 1.941 | 1.253 |
| Cu | 8.491259 | 3.039 | 65.65667 | 6.120667 |
| Fe | 202.9381 | 89.85667 | 699.9333 | 176.0667 |
| Ga | 0.254843 | 0.0367 | 0.5593 | 0.233233 |
| Ge | 0.200248 | 0.0238 | 0.55195 | 0.1767 |
| Hg | 0.117044 | 0.0348 | 0.211733 | 0.116233 |
| In | 0.25141 | 0.047433 | 0.571533 | 0.2507 |
| K | 4016.455 | 2822.333 | 5374.667 | 4015.333 |
| Li | 0.020301 | 0.0009 | 0.089067 | 0.020367 |
| Mg | 349.4238 | 271.2333 | 505.5667 | 346.7667 |
| Mn | 8.572944 | 1.732333 | 14.50667 | 8.723667 |
| Mo | 1.39697 | 0.0855 | 3.468333 | 1.184667 |
| Na | 1149.126 | 472.6667 | 2602.333 | 1132.667 |
| Nb | 0.302752 | 0.042167 | 0.4664 | 0.317033 |
| Ni | 0.610432 | 0.3808 | 0.8783 | 0.61425 |
| P | 9047.55 | 6916 | 12936.67 | 9034.667 |
| Pb | 0.235276 | 0.052833 | 0.41705 | 0.22965 |
| Rb | 9.158524 | 3.787667 | 21.63 | 8.676 |
| S | 5270.659 | 4235.667 | 7430.333 | 5158 |
| Sb | 0.133119 | 0.023767 | 0.324067 | 0.104967 |
| Se | 0.599884 | 0.105133 | 1.923667 | 0.5376 |
| Si | 1.532586 | 1.04 | 2.488 | 1.455 |
| Sn | 0.037502 | 0.011767 | 0.0783 | 0.03505 |
| Sr | 0.351094 | 0.172733 | 0.877083 | 0.333 |
| Ta | 0.216474 | 0.044967 | 0.4337 | 0.2119 |
| Ti | 0.173298 | 0.067467 | 0.808367 | 0.142933 |
| Tl | 0.143125 | 0.02615 | 0.327133 | 0.135033 |
| V | 1.119797 | 0.2875 | 1.693 | 1.100667 |
| W | 0.143288 | 0.00175 | 0.4088 | 0.1143 |
| Zn | 100.9064 | 32.21 | 627.5667 | 73.3 |
| Zr | 0.032355 | 0.003967 | 0.062233 | 0.0314 |
