# Supplementary material for: Comorbidities and concentration of trace elements in livers of European bison from Bieszczady Mountains (Poland)
Source: Sci Rep. 2023 Mar 15;13:4332. doi: 10.1038/s41598-023-31245-z (PMC10017800; doi:10.1038/s41598-023-31245-z)
Supplement: Supplementary file 5 — Supplementary Table S5. [file 41598_2023_31245_MOESM5_ESM.docx]

Table S5. Pearson correlation matrix for the concentrations of the elements studied; the values of the Pearson correlation coefficient (r) and probability (p) for each element pair are given. Statistically significant values (p <0.05) are highlighted in red.

|  |  | Al | As | Ca | Cd | Co | Cu | Fe | Hg | Li | Mg | Mn | Mo | Ni | Pb | Se | Sn | Ti | V | Zn |
| --- | --- | --- | --- | --- | --- | --- | --- | --- | --- | --- | --- | --- | --- | --- | --- | --- | --- | --- | --- | --- |
| Al | r | 1 | 0.120 | 0.119 | 0.194 | 0.059 | 0.105 | 0.182 | 0.098 | 0.097 | -0.221 | -0.209 | 0.016 | -0.076 | -0.108 | 0.108 | 0.015 | 0.050 | -0.030 | 0.480 |
|  | p |  | 0.348 | 0.352 | 0.127 | 0.644 | 0.414 | 0.153 | 0.443 | 0.449 | 0.081 | 0.101 | 0.901 | 0.552 | 0.400 | 0.398 | 0.904 | 0.696 | 0.813 | <0.001 |
| As | r | 0.120 | 1 | 0.149 | 0.079 | -0.113 | 0.092 | 0.037 | 0.371 | 0.161 | 0.082 | -0.086 | 0.013 | 0.026 | 0.166 | -0.457 | -0.051 | 0.037 | 0.407 | 0.036 |
|  | p | 0.348 |  | 0.243 | 0.540 | 0.377 | 0.473 | 0.774 | 0.003 | 0.207 | 0.521 | 0.501 | 0.920 | 0.837 | 0.194 | <0.001 | 0.689 | 0.773 | <0.001 | 0.782 |
| Ca | r | 0.119 | 0.149 | 1 | 0.377 | 0.132 | -0.192 | 0.040 | 0.113 | -0.172 | 0.425 | 0.015 | 0.054 | 0.314 | 0.067 | 0.277 | -0.094 | 0.036 | 0.294 | 0.280 |
|  | p | 0.352 | 0.243 |  | 0.002 | 0.303 | 0.132 | 0.754 | 0.379 | 0.177 | <0.001 | 0.906 | 0.672 | 0.012 | 0.602 | 0.028 | 0.464 | 0.777 | 0.019 | 0.026 |
| Cd | r | 0.194 | 0.079 | 0.377 | 1 | -0.050 | 0.130 | 0.233 | 0.073 | -0.018 | 0.180 | -0.142 | -0.062 | -0.052 | 0.044 | 0.459 | -0.111 | -0.030 | 0.234 | -0.005 |
|  | p | 0.127 | 0.540 | 0.002 |  | 0.700 | 0.310 | 0.066 | 0.572 | 0.887 | 0.158 | 0.266 | 0.628 | 0.686 | 0.732 | <0.001 | 0.387 | 0.817 | 0.064 | 0.971 |
| Co | r | 0.059 | -0.113 | 0.132 | -0.050 | 1 | -0.032 | 0.000 | -0.316 | 0.142 | -0.136 | 0.218 | -0.043 | 0.259 | 0.066 | 0.171 | 0.106 | 0.115 | -0.057 | 0.304 |
|  | p | 0.644 | 0.377 | 0.303 | 0.700 |  | 0.802 | 0.998 | 0.012 | 0.267 | 0.288 | 0.086 | 0.739 | 0.041 | 0.608 | 0.180 | 0.408 | 0.371 | 0.657 | 0.015 |
| Cu | r | 0.105 | 0.092 | -0.192 | 0.130 | -0.032 | 1 | -0.089 | 0.124 | 0.044 | -0.169 | -0.310 | 0.152 | -0.228 | -0.061 | -0.100 | -0.276 | 0.418 | -0.016 | -0.068 |
|  | p | 0.414 | 0.473 | 0.132 | 0.310 | 0.802 |  | 0.489 | 0.335 | 0.734 | 0.185 | 0.013 | 0.234 | 0.072 | 0.635 | 0.437 | 0.029 | <0.001 | 0.903 | 0.595 |
| Fe | r | 0.182 | 0.037 | 0.040 | 0.233 | 0.000 | -0.089 | 1 | 0.099 | 0.139 | -0.093 | -0.088 | 0.069 | -0.209 | 0.204 | 0.021 | 0.117 | -0.179 | -0.227 | 0.083 |
|  | p | 0.153 | 0.774 | 0.754 | 0.066 | 0.998 | 0.489 |  | 0.441 | 0.277 | 0.467 | 0.494 | 0.592 | 0.101 | 0.109 | 0.873 | 0.359 | 0.160 | 0.074 | 0.518 |
| Hg | r | 0.098 | 0.371 | 0.113 | 0.073 | -0.316 | 0.124 | 0.099 | 1 | 0.246 | -0.038 | -0.173 | 0.267 | -0.220 | -0.013 | -0.358 | 0.026 | 0.027 | 0.175 | -0.040 |
|  | p | 0.443 | 0.003 | 0.379 | 0.572 | 0.012 | 0.335 | 0.441 |  | 0.052 | 0.768 | 0.175 | 0.035 | 0.083 | 0.917 | 0.004 | 0.839 | 0.831 | 0.170 | 0.758 |
| Li | r | 0.097 | 0.161 | -0.172 | -0.018 | 0.142 | 0.044 | 0.139 | 0.246 | 1 | -0.225 | -0.092 | 0.212 | -0.161 | 0.186 | -0.253 | 0.214 | -0.133 | -0.073 | 0.093 |
|  | p | 0.449 | 0.207 | 0.177 | 0.887 | 0.267 | 0.734 | 0.277 | 0.052 |  | 0.076 | 0.475 | 0.096 | 0.209 | 0.144 | 0.046 | 0.092 | 0.298 | 0.569 | 0.470 |
| Mg | r | -0.221 | 0.082 | 0.425 | 0.180 | -0.136 | -0.169 | -0.093 | -0.038 | -0.225 | 1 | -0.002 | -0.003 | 0.355 | 0.009 | 0.262 | -0.161 | 0.212 | 0.243 | -0.102 |
|  | p | 0.081 | 0.521 | <0.001 | 0.158 | 0.288 | 0.185 | 0.467 | 0.768 | 0.076 |  | 0.986 | 0.982 | 0.004 | 0.947 | 0.038 | 0.208 | 0.096 | 0.055 | 0.427 |
| Mn | r | -0.209 | -0.086 | 0.015 | -0.142 | 0.218 | -0.310 | -0.088 | -0.173 | -0.092 | -0.002 | 1 | 0.089 | 0.261 | 0.248 | 0.055 | -0.012 | 0.014 | 0.197 | 0.044 |
|  | p | 0.101 | 0.501 | 0.906 | 0.266 | 0.086 | 0.013 | 0.494 | 0.175 | 0.475 | 0.986 |  | 0.489 | 0.039 | 0.050 | 0.669 | 0.927 | 0.912 | 0.122 | 0.733 |
| Mo | r | 0.016 | 0.013 | 0.054 | -0.062 | -0.043 | 0.152 | 0.069 | 0.267 | 0.212 | -0.003 | 0.089 | 1 | 0.055 | 0.193 | -0.080 | -0.148 | 0.261 | 0.155 | -0.064 |
|  | p | 0.901 | 0.920 | 0.672 | 0.628 | 0.739 | 0.234 | 0.592 | 0.035 | 0.096 | 0.982 | 0.489 |  | 0.669 | 0.129 | 0.535 | 0.247 | 0.039 | 0.224 | 0.620 |
| Ni | r | -0.076 | 0.026 | 0.314 | -0.052 | 0.259 | -0.228 | -0.209 | -0.220 | -0.161 | 0.355 | 0.261 | 0.055 | 1 | 0.139 | 0.185 | -0.206 | 0.156 | 0.288 | 0.052 |
|  | p | 0.552 | 0.837 | 0.012 | 0.686 | 0.041 | 0.072 | 0.101 | 0.083 | 0.209 | 0.004 | 0.039 | 0.669 |  | 0.278 | 0.147 | 0.106 | 0.221 | 0.022 | 0.684 |
| Pb | r | -0.108 | 0.166 | 0.067 | 0.044 | 0.066 | -0.061 | 0.204 | -0.013 | 0.186 | 0.009 | 0.248* | 0.193 | 0.139 | 1 | -0.101 | -0.279 | 0.006 | 0.161 | -0.188 |
|  | p | 0.400 | 0.194 | 0.602 | 0.732 | 0.608 | 0.635 | 0.109 | 0.917 | 0.144 | 0.947 | 0.050 | 0.129 | 0.278 |  | 0.431 | 0.027 | 0.964 | 0.207 | 0.140 |
| Se | r | 0.108 | -0.457 | 0.277 | 0.459 | 0.171 | -0.100 | 0.021 | -0.358 | -0.253 | 0.262 | 0.055 | -0.080 | 0.185 | -0.101 | 1 | 0.045 | 0.129 | -0.116 | -0.039 |
|  | p | 0.398 | <0.001 | 0.028 | <0.001 | 0.180 | 0.437 | 0.873 | 0.004 | 0.046 | 0.038 | 0.669 | 0.535 | 0.147 | 0.431 |  | 0.725 | 0.313 | 0.365 | 0.763 |
| Sn | r | 0.015 | -0.051 | -0.094 | -0.111 | 0.106 | -0.276 | 0.117 | 0.026 | 0.214 | -0.161 | -0.012 | -0.148 | -0.206 | -0.279 | 0.045 | 1 | -0.244 | -0.303 | 0.143 |
|  | p | 0.904 | 0.689 | 0.464 | 0.387 | 0.408 | 0.029 | 0.359 | 0.839 | 0.092 | 0.208 | 0.927 | 0.247 | 0.106 | 0.027 | 0.725 |  | 0.054 | 0.016 | 0.265 |
| Ti | r | 0.050 | 0.037 | 0.036 | -0.030 | 0.115 | 0.418 | -0.179 | 0.027 | -0.133 | 0.212 | 0.014 | 0.261 | 0.156 | 0.006 | 0.129 | -0.244 | 1 | 0.112 | -0.070 |
|  | p | 0.696 | 0.773 | 0.777 | 0.817 | 0.371 | <0.001 | 0.160 | 0.831 | 0.298 | 0.096 | 0.912 | 0.039 | 0.221 | 0.964 | 0.313 | 0.054 |  | 0.381 | 0.585 |
| V | r | -0.030 | 0.407 | 0.294 | 0.234 | -0.057 | -0.016 | -0.227 | 0.175 | -0.073 | 0.243 | 0.197 | 0.155 | 0.288 | 0.161 | -0.116 | -0.303 | 0.112 | 1 | 0.069 |
|  | p | 0.813 | <0.001 | 0.019 | 0.064 | 0.657 | 0.903 | 0.074 | 0.170 | 0.569 | 0.055 | 0.122 | 0.224 | 0.022 | 0.207 | 0.365 | 0.016 | 0.381 |  | 0.593 |
| Zn | r | 0.480 | 0.036 | 0.280 | -0.005 | 0.304 | -0.068 | 0.083 | -0.040 | 0.093 | -0.102 | 0.044 | -0.064 | 0.052 | -0.188 | -0.039 | 0.143 | -0.070 | 0.069 | 1 |
|  | p | <0.001 | 0.782 | 0.026 | 0.971 | 0.015 | 0.595 | 0.518 | 0.758 | 0.470 | 0.427 | 0.733 | 0.620 | 0.684 | 0.140 | 0.763 | 0.265 | 0.585 | 0.593 |  |
