# Supplementary material for: Comorbidities and concentration of trace elements in livers of European bison from Bieszczady Mountains (Poland)
Source: Sci Rep. 2023 Mar 15;13:4332. doi: 10.1038/s41598-023-31245-z (PMC10017800; doi:10.1038/s41598-023-31245-z)
Supplement: Supplementary file 6 — Supplementary Table S6. [file 41598_2023_31245_MOESM6_ESM.docx]

Table S3. Effect of age, sex and comorbidities on hepatic concentration of given element (in table Wald Χ^2^ test or t test was given depending on the model used: generalized linear model (GzLM) or general linear model (GLM) respectively).

| Element (model type) | Source | B | Se | Wald Χ^2^/t | p |
| --- | --- | --- | --- | --- | --- |
| Al (GLM) | Intercept | 2.007 | 0.230 | 8.717 | <0.001 |
|  | Age | 0.004 | 0.015 | 0.250 | 0.803 |
|  | Sex (F) | -0.035 | 0.132 | -0.264 | 0.793 |
|  | Disease (Group A) | -0.188 | 0.194 | -0.969 | 0.337 |
|  | Disease (Group B) | -0.190 | 0.191 | -0.997 | 0.323 |
|  |  |  |  |  |  |
| As (GLM) | Intercept | 0.301 | 0.045 | 6.668 | <0.001 |
|  | Age | -0.001 | 0.003 | -0.196 | 0.845 |
|  | Sex (F) | 0.003 | 0.026 | 0.115 | 0.909 |
|  | Disease (Group A) | -0.010 | 0.038 | -0.262 | 0.794 |
|  | Disease (Group B) | -0.020 | 0.037 | -0.545 | 0.588 |
|  |  |  |  |  |  |
| Ca (GLM) | Intercept | 132.651 | 10.584 | 12.533 | <0.001 |
|  | Age | -0.373 | 0.694 | -0.538 | 0.593 |
|  | Sex (F) | -16.128 | 6.057 | -2.663 | 0.010 |
|  | Disease (Group A) | 4.255 | 8.900 | 0.478 | 0.634 |
|  | Disease (Group B) | 6.854 | 8.768 | 0.782 | 0.438 |
|  |  |  |  |  |  |
| Cd (GzLM) | Intercept | 0.363 | 0.279 | 1.687 | 0.194 |
|  | Age | 0.061 | 0.019 | 10.841 | <0.001 |
|  | Sex (F) | -0.013 | 0.173 | 0.006 | 0.940 |
|  | Disease (Group A) | -0.323 | 0.247 | 1.711 | 0.191 |
|  | Disease (Group B) | -0.214 | 0.246 | 0.756 | 0.384 |
|  |  |  |  |  |  |
| Co (GLM) | Intercept | 0.142 | 0.012 | 11.757 | <0.001 |
|  | Age | -0.002 | 0.001 | -1.926 | 0.059 |
|  | Sex (F) | -0.007 | 0.007 | -1.080 | 0.285 |
|  | Disease (Group A) | -0.018 | 0.010 | -1.771 | 0.082 |
|  | Disease (Group B) | -0.008 | 0.010 | -0.767 | 0.446 |
|  |  |  |  |  |  |
| Cu (GzLM) | Intercept | 2.140 | 0.270 | 62.836 | <0.001 |
|  | Age | 0.004 | 0.019 | 0.041 | 0.840 |
|  | Sex (F) | -0.634 | 0.148 | 18.424 | <0.001 |
|  | Disease (Group A) | 0.446 | 0.212 | 4.403 | 0.036 |
|  | Disease (Group B) | -0.063 | 0.214 | 0.087 | 0.768 |
|  |  |  |  |  |  |
| Fe (GLM) | Intercept | 2.519 | 0.037 | 68.098 | <0.001 |
|  | Age | 0.002 | 0.002 | 0.893 | 0.376 |
|  | Sex (F) | -0.002 | 0.021 | -0.117 | 0.908 |
|  | Disease (Group A) | -0.014 | 0.031 | -0.447 | 0.657 |
|  | Disease (Group B) | 0.008 | 0.031 | 0.264 | 0.793 |
|  |  |  |  |  |  |
| Hg (GLM) | Intercept | 0.111 | 0.015 | 7.313 | <0.001 |
|  | Age | 0.000 | 0.001 | -0.278 | 0.782 |
|  | Sex (F) | -0.008 | 0.009 | -0.884 | 0.380 |
|  | Disease (Group A) | 0.023 | 0.013 | 1.833 | 0.072 |
|  | Disease (Group B) | 0.007 | 0.013 | 0.574 | 0.568 |
|  |  |  |  |  |  |
| Li (GLM) | Intercept | -2.135 | 0.085 | -25.048 | <0.001 |
|  | Age | 0.006 | 0.006 | 1.046 | 0.300 |
|  | Sex (F) | -0.036 | 0.049 | -0.736 | 0.465 |
|  | Disease (Group A) | 0.033 | 0.072 | 0.466 | 0.643 |
|  | Disease (Group B) | -0.078 | 0.071 | -1.102 | 0.064 |
|  |  |  |  |  |  |
| Mg (GLM) | Intercept | 371.627 | 20.631 | 18.013 | <0.001 |
|  | Age | -1.698 | 1.352 | -1.255 | 0.214 |
|  | Sex (F) | -27.982 | 11.805 | -2.370 | 0.021 |
|  | Disease (Group A) | -1.385 | 17.348 | -0.080 | 0.937 |
|  | Disease (Group B) | 25.024 | 17.091 | 1.464 | 0.149 |
|  |  |  |  |  |  |
| Mn (GLM) | Intercept | 9.271 | 0.999 | 9.278 | <0.001 |
|  | Age | -0.081 | 0.065 | -1.233 | 0.223 |
|  | Sex (F) | 1.122 | 0.572 | 1.963 | 0.055 |
|  | Disease (Group A) | -0.874 | 0.840 | -1.040 | 0.303 |
|  | Disease (Group B) | -0.315 | 0.828 | -0.380 | 0.705 |
|  |  |  |  |  |  |
| Mo (GLM) | Intercept | 0.171 | 0.283 | 0.604 | 0.548 |
|  | Age | 0.014 | 0.019 | 0.771 | 0.444 |
|  | Sex (F) | -0.366 | 0.162 | -2.261 | 0.028 |
|  | Disease (Group A) | 0.200 | 0.238 | 0.842 | 0.403 |
|  | Disease (Group B) | -0.010 | 0.234 | -0.042 | 0.966 |
|  |  |  |  |  |  |
| Ni (GLM) | Intercept | 0.798 | 0.043 | 18.168 | <0.001 |
|  | Age | -0.010 | 0.003 | -3.429 | 0.001 |
|  | Sex (F) | -0.057 | 0.025 | -2.285 | 0.026 |
|  | Disease (Group A) | -0.063 | 0.037 | -1.737 | 0.088 |
|  | Disease (Group B) | -0.026 | 0.036 | -0.724 | 0.472 |
|  |  |  |  |  |  |
| Pb (GLM) | Intercept | 0.255 | 0.047 | 5.419 | <0.001 |
|  | Age | 0.000 | 0.003 | 0.018 | 0.986 |
|  | Sex (F) | 0.019 | 0.027 | 0.690 | 0.493 |
|  | Disease (Group A) | -0.020 | 0.040 | -0.511 | 0.611 |
|  | Disease (Group B) | -0.047 | 0.039 | -1.193 | 0.238 |
|  |  |  |  |  |  |
| Se (GLM) | Intercept | 0.609 | 0.147 | 4.1136 | <0.001 |
|  | Age | 0.010 | 0.010 | 1.073 | 0.288 |
|  | Sex (F) | -0.023 | 0.084 | -0.273 | 0.785 |
|  | Disease (Group A) | -0.274 | 0.124 | -2.210 | 0.031 |
|  | Disease (Group B) | 0.031 | 0.122 | -0.256 | 0.799 |
|  |  |  |  |  |  |
| Sn (GLM) | Intercept | -2.296 | 0.095 | -24.112 | <0.001 |
|  | Age | 0.003 | 0.006 | 0.440 | 0.662 |
|  | Sex (F) | 0.057 | 0.054 | 1.055 | 0.296 |
|  | Disease (Group A) | -0.113 | 0.080 | -1.412 | 0.164 |
|  | Disease (Group B) | -0.119 | 0.079 | -1.509 | 0.137 |
|  |  |  |  |  |  |
| Ti (GLM) | Intercept | -2.992 | 0.724 | -4.131 | <0.001 |
|  | Age | -0.033 | 0.047 | -0.685 | 0.496 |
|  | Sex (F) | -0.766 | 0.414 | -1.847 | 0.070 |
|  | Disease (Group A) | -0.299 | 0.609 | -0.491 | 0.625 |
|  | Disease (Group B) | -0.207 | 0.600 | -0.354 | 0.731 |
|  |  |  |  |  |  |
| V (GLM) | Intercept | 1.187 | 0.152 | 7.836 | <0.001 |
|  | Age | -0.012 | 0.010 | -1.174 | 0.245 |
|  | Sex (F) | 0.000 | 0.087 | 0.001 | 0.999 |
|  | Disease (Group A) | 0.068 | 0.127 | 0.533 | 0.596 |
|  | Disease (Group B) | 0.098 | 0.126 | 0.777 | 0.440 |
|  |  |  |  |  |  |
| Zn (GzLM) | Intercept | 4.655 | 0.247 | 356.586 | 0.000 |
|  | Age | 0.013 | 0.165 | 0.620 | 0.431 |
|  | Sex (F) | -0.431 | 0.143 | 9.125 | 0.003 |
|  | Disease (Group A) | -0.245 | 0.211 | 1.351 | 0.245 |
|  | Disease (Group B) | 0.111 | 0.208 | 0.286 | 0.593 |
